# Impacts of the COVID-19 Pandemic on United States Emergency Medicine Education: A Council of Residency Directors in Emergency Medicine (CORD) Task Force Survey-Based Analysis

**DOI:** 10.7759/cureus.35994

**Published:** 2023-03-10

**Authors:** Sarah Dunn, Brian D Milman, Rebecca A Bavolek, Leah Bralow, David Jones, Bryan G Kane, Stephen Miller, Shannon Moffett, Lisa Stoneking, Morgan D Wilbanks, Melissa A Platt

**Affiliations:** 1 Emergency Medicine, Rutgers University New Jersey Medical School, Newark, USA; 2 Emergency Medicine, University of Oklahoma School of Community Medicine, Tulsa, USA; 3 Emergency Medicine, University of California Los Angeles David Geffen School of Medicine, Los Angeles, USA; 4 Emergency Medicine, St. Barnabas Hospital Health System, Bronx, USA; 5 Emergency Medicine, Oregon Health & Science University, Portland, USA; 6 Emergency Medicine and Hospital Medicine, Lehigh Valley Health Network, Allentown, USA; 7 Emergency Medicine, Virginia Commonwealth University (VCU) Medical Center, Richmond, USA; 8 Emergency Medicine, University of Arizona, Tucson, USA; 9 Emergency Medicine, Medical College of Wisconsin, Milwaukee, USA; 10 Emergency Medicine, University of Louisville, Louisville, USA

**Keywords:** virtual teaching, emergency medicine training, teaching in emergency medicine, covid-19 pandemic, covid-19

## Abstract

Introduction

The COVID-19 pandemic presented unpredicted challenges to Emergency Medicine (EM) education. The rapid onset of the pandemic created clinical, operational, administrative, and home-life challenges for virtually every member of the medical education community, demanding an educational and professional response at all levels including undergraduate medical education (UME), graduate medical education (GME), and faculty. The Council of Residency Directors in Emergency Medicine (CORD) COVID-19 Educational Impact Task Force was established in 2021 to examine these effects and the response of the EM educational community.

Methods

The Task Force utilized consensus methodology to develop the survey instruments, which were revised using a modified Delphi process. Both open- and closed-answer questions were included in the survey, which was initially distributed electronically to attendees of the 2021 Virtual Academic Assembly. Results were analyzed quantitatively and qualitatively.

Results

Sixty-three individuals responded to the first part of the survey (which addressed issues related to UME and GME) and 41 individuals responded to the second part of the survey (which addressed faculty and wellness). The pandemic’s influence on EM education was viewed in both a positive and negative light. The transition to virtual platforms had various impacts, including innovation and engagement via technology. Remote technology improved participation in didactics and allowed individuals to more easily participate in departmental meetings. However, this also led to a decreased sense of connection with peers and colleagues resulting in a mixed picture for overall engagement and effectiveness. The Task Force has developed a list of recommendations for best practices for EM programs and for EM organizations.

Conclusion

The survey results articulated the educational benefits and challenges faced by EM educators during the COVID-19 pandemic. Through the challenging times of the pandemic, many institutional and program-based innovations were developed and implemented to address the new educational environment. These approaches will provide invaluable educational tools for future training. This will also prepare the EM academic community to respond to future educational disruptions.

## Introduction

The COVID-19 pandemic has presented unpredicted challenges to Emergency Medicine (EM) education. The rapid onset of the pandemic created clinical, operational, administrative, and home-life challenges for virtually every member of the medical education community. This unprecedented event demanded an educational and professional response at all levels. Effects were felt in undergraduate medical education (UME), the Match process, graduate medical education (GME), faculty development, and wellness [1-4]. The Council of Residency Directors in Emergency Medicine (CORD) COVID-19 Educational Impact Task Force was established in 2021 to examine these effects and the response of the EM educational community [5].

Through the challenging times of the pandemic, many institutional and program-based innovations were developed and implemented to address education barriers [6]. While individual endeavors were key to overcoming educational constraints, there was no clear coordinated effort within academic EM to examine these endeavors. In order to do this, the CORD Board of Directors convened the COVID-19 Educational Task Force in February 2021 with a charter to investigate the impact of the COVID-19 pandemic on EM education [5]. Membership on the Task Force was determined by an application process and the Task Force first convened in February 2021 to discuss this goal. The Task Force moved quickly to develop a survey to collect data related to experiences during the pandemic as well as an initial needs assessment. The goal was to capture the educational experiences during the pandemic in order to create a roadmap for future endeavors and explore how organizations such as CORD could support their members during similar events in the future.

## Materials and methods

The Task Force utilized consensus methodology to develop the survey instruments [7]. Topic areas were identified using expert consensus and revised using a modified Delphi process [8]. A sub-group of the Task Force developed a draft survey which was then iteratively developed using three cycles of change. Both open- and closed-answer questions were included in the survey, with the former occurring first in order to increase construct validity [8]. Similarly, demographics were entered at the end in order to limit the introduction of bias [9]. To address criterion issues, previously validated means of response were utilized when possible [8]. The pilot version of the survey was distributed to the remaining Task Force members and revised for face validity [8]. Prior to distribution, the project was reviewed and approved by the University of Louisville IRB #21.0323.

The final survey was distributed electronically to attendees of the 2021 Virtual Academic Assembly, held April 11-15, 2021. To improve participation and completion, the survey was conducted in two parts [10]. Part 1 examined the impact of the pandemic on UME and GME. Part 2 examined the impact on faculty and on personal wellness. After the Academic Assembly, the survey was also distributed electronically to CORD membership via the subscribed CORD survey listserv. This survey was subsequently distributed again in May of 2021 to improve the completion rate, and the available survey was also highlighted in the July 2021 CORD electronic newsletter.

Analysis was both quantitative and qualitative. The quantitative analysis was performed using Statistical Product and Service Solutions (SPSS) (IBM SPSS Statistics for Windows, Version 26.0, Armonk, NY). The quantitative analysis was descriptive. We employed qualitative thematic coding mechanisms for all open-ended questions [11]. This analysis was descriptive and utilized the iterative development of a codebook [12]. Over four cycles, the group thematically analyzed the responses to the open-ended questions. Through this iterative process, responses were grouped thematically with the last cycle resulting in the coding of no more than three thematic categories per response. The presentation of qualitative results is primarily descriptive. Quotations were selected to communicate examples of the central themes for each category.

## Results

Survey part 1 - UME and GME

Description of Respondents

Sixty-three individuals responded to the first part of the survey, with 27 (42.9%) program directors (PDs), 19 (30.2%) assistant/associate PDs, five (7.9%) core faculty, five (7.9%) clerkship directors, four (6.3%) residents/fellows, and three others (vice chair of education, educational researcher, unknown). Most respondents were white (84.1%) and approximately half identified as women (50.8%).

UME Quantitative Results

Benefits and challenges were identified by survey respondents (Table 1). The highest-ranked benefit was the reduced financial burden related to decreased travel for rotations and interviews. Other highly ranked benefits included the use of virtual platforms and new educational modalities including asynchronous learning. Survey respondents felt that students having restrictions on clinical experiences was the most important challenge faced during this time period.

**Table 1:** UME Quantitative Results UME: undergraduate medical education

Item	Mean	SD
UME Benefits - Rank 1 to 6 with 1 being the most important.		
Decreased financial burden of away rotations/interviews	2.53	1.76
Increased utilization of asynchronous learning	3.08	1.49
Use of videoconferencing programs (Zoom, etc.)	3.29	1.61
Re-evaluation of current education modalities for students	3.63	1.68
Ability to attend virtual education sessions from a variety of departments/programs	3.69	1.58
Time for students to participate in scholarly activity	4.77	1.29
UME Challenges - Rank 1 to 7 with 1 being the most important.		
Students pulled from clinical rotations	1.40	0.88
How students get the “fit” of the program over the virtual platform	3.32	1.61
Use of virtual rotations while students were pulled from clinical experiences	4.18	1.47
Restrictions on simulation activities	4.45	1.73
Inability to host an in-person lecture	4.58	1.65
Virtual interviews	4.70	2.00
Students having to remediate required clinical rotations prior to fourth-year electives	5.30	1.77

UME Qualitative Results

Analysis of the open-ended survey responses demonstrated several themes which were similar to those reflected in the ranking questions (Table 2). Benefits included changes to the teaching experience, the use of remote platforms, and financial benefits. Overwhelmingly, the greatest challenges named by respondents were related to diminished clinical and educational experiences. Respondents also commented frequently on the negative impact on the acquisition of medical skills and knowledge and on the loss of personal connection.

**Table 2:** UME Qualitative Results UME: undergraduate medical education; EM: emergency medicine

Benefits (119 responses)	
Theme (% of responses)	Examples of Individual Responses
Teaching experience (20%)	Broadened the reach for most students with virtual platforms; creation of innovative electives to replace clinical time
Remote (19%)	Easier at times to meet with an advisor virtually; increased ability to review programs and meet virtually with program faculty
Financial (18%)	Decreased travel cost of interviews; less money spent on away rotations
Technology (16%)	The increased utilization of virtual platforms for expert speakers; Access to recorded sessions
Skills and knowledge (11%)	Direct learning about pandemic response; learning operational skills to gather and allocate resources
Challenges (161 responses)	
Theme (% of responses)	Examples of Individual Responses
Clinical Experiences (60%)	Education of medical students without a clinical environment; unable to do a second EM rotation to show improvement
Cancellations/Scheduling (45%)	Delayed or canceled rotations; fewer total rotations prior to the Match
Skills and Knowledge (26%)	Concentration of COVID-19 with loss of other content; lack of hands-on experience; virtual classes put together at a moment’s notice
Personal Connection (18%)	Development of professional interpersonal relationships, support networks, and community
Recruitment (17%)	Difficulty getting applicants to see the residency program; stress and risk of inequalities of the virtual interview process

GME Quantitative Results

Major benefits were improved faculty attendance and participation in virtual conferences, educational innovation, and easier attendance for virtual committee meetings (Table 3). Other benefits were related to new educational modalities, including the decreased financial burden of using virtual platforms. The most prominent challenges facing GME during the pandemic were loss of personal educational interactions, difficulty for residents to form bonds with peers, and diminished learner participation during conferences.

**Table 3:** GME Quantitative Results GME: graduate medical education

Item	Mean	SD
GME Benefits - Rank 1 to 11 with 1 being the most important.		
Improved faculty attendance at a virtual conference	3.00	2.15
Educational innovation	3.17	1.97
Easier attendance for virtual committee meetings (departmental and institutional)	3.75	2.43
Ability to have multi-institutional conferences via video conferencing	4.24	2.83
Increased asynchronous learning	5.25	2.27
Decreased financial burden for grand rounds speakers	6.31	2.99
Recorded conference lectures/created a lecture library	6.38	2.56
Decreased financial burden for conference food	8.04	2.56
Stress and wellness management	8.05	2.58
Virtual mentorship	8.34	1.88
Virtual support system	9.00	2.07
GME Challenges - Rank 1 to 10 with 1 being the most important.		
Loss of personal, face-to-face educational interactions.	2.74	2.07
Forming bonds with peers	3.30	1.94
Changes in learner participation during the conference	4.47	2.26
Stress and wellness management	4.71	2.72
Reduced ability to use simulation lab	4.98	2.43
Ensuring continued high-quality educational content	5.22	2.65
Participation in small group learning	6.15	2.05
Video conferencing platform	6.71	2.72
Virtual mentorship	8.11	1.64
Virtual support system	8.14	2.45

GME Qualitative Results

GME members also provided substantial qualitative responses regarding the pandemic’s impact on training EM residents (Table 4). Many felt that the transition to remote learning platforms as well as innovation in the clinical environment was beneficial to EM resident learners. Twenty-six percent of respondents perceived an improvement in skills and knowledge, frequently in the areas of airway management, critical care, and disaster preparedness. Challenges in GME included impacts on wellness, disrupted clinical experiences, and loss of personal connection.

**Table 4:** GME Qualitative Results GME: graduate medical education; PTSD: post-traumatic stress disorder; ICU: intensive care unit; EM: emergency medicine

Benefits (103 total responses)	
Theme (% of responses)	Examples of Individual Responses
Skills and knowledge (26%)	Opportunity to learn clinical operations and disaster medicine skills; learning safer ways to do procedures
Remote (22%)	Able to attend teaching and meetings remotely in order to maximize time off
Clinical experience (19%)	Educational experience of rapid changes in how to treat patients in the pandemic; increased critical care time
Teaching experience (18%)	Incorporation of new educational technologies/educational innovation
Innovation (17%)	Innovations in the curriculum; innovative airway management
Challenges (156 total responses)	
Theme (% of responses)	Examples of Individual Responses
Wellness (30%)	Harder to get the temperature of residents/residency as far as well-being; managing residents with PTSD, depression, burnout, etc. related to and exacerbated by COVID-19
Cancellations/ scheduling (22%)	All services pulled into ICUs/COVID-19 units; cancelled rotations- anesthesia
Skills and Knowledge (22%)	Concentration on COVID-19 has reduced exposure and learning about other disease processes
Loss of personal connection (21%)	Loss of GME-sponsored 'fun' activities - dinners, graduation, golf outing
Clinical experiences (19%)	Decreased clinical exposure to important skills that are essential for EM but not frequently encountered in the department (i.e., deliveries); loss of breadth of clinical experience

Survey part 2 - Faculty and personal wellness

Description of Respondents

Forty-one individuals responded to the second part of the survey. Eighteen (43.9%) respondents were PDs, 14 (34.1%) were assistant/associate PDs, four (9.8%) were core faculty, four (9.8%) were clerkship directors, and one individual (2.4%) was involved with faculty development and research. Most respondents were white (87.8%) and women (61%).

Quantitative Results

As the majority (98%) of respondents were faculty, the faculty and personal wellness responses were combined below (Table 5). Faculty noted the educational benefits and challenges of the new educational environment. Benefits included increased levels of faculty engagement, educational innovation, increased participation in committees, and financial savings. However, faculty also encountered challenges when adapting to the near-total virtual educational environment. The loss of personal connectivity and forming bonds with others was the main faculty challenge. On a personal level, respondents generally felt supported during the pandemic by family, colleagues, and the community and reported an ability to self-reflect. However, they simultaneously noted stress, burnout, and isolation as significant challenges.

**Table 5:** Faculty and Wellness Quantitative Results

Item	Mean	SD
Faculty Educational Benefits - Rank 1 to 8 with 1 being the most important.		
Faculty engagement in residency conference	2.24	1.48
Virtual video conference platform	2.68	1.65
Educational innovation	3.97	2.38
Faculty involvement in committees	4.27	1.77
Committee meeting attendance	4.43	2.13
Decreased financial burden on departments for faculty development programs/speakers	5.65	2.47
Recorded lectures were given by faculty	6.00	1.43
Recorded faculty development sessions	6.11	1.32
Faculty Educational Challenges - Rank 1 to 6 with 1 being the most important.		
Forming bonds with peers/residents	1.56	0.88
Faculty engagement in the resident conference	3.62	1.70
Virtual mentorship	3.66	1.77
Repurposing faculty into virtual roles	3.87	1.73
Faculty engagement in departmental meetings/committees	3.89	1.03
Virtual video conference platform	4.35	1.48
Wellness Benefits - Rank 1 to 8 with 1 being the most important.		
Support of family/friends	2.29	1.41
Self-reflection or realization	2.61	1.59
Departmental support	3.63	2.22
Focus on physical and mental health	3.94	1.69
Support from the public - e.g., acts of recognition for frontline workers	4.89	1.75
Food donations to the department for frontline workers.	5.85	1.94
Departmental programming on stress management	6.18	1.42
Decreased financial burden from financial forgiveness programs	6.18	2.21
Wellness Challenges - Rank 1 to 9 with 1 being the most important.		
Stress and wellness management	3.60	2.03
Psychological distress	3.75	2.50
Burnout	4.17	2.36
Forming bonds with peers	4.30	2.65
Loss of non-clinical support systems (friends/family) due to distancing	4.58	2.68
Feelings of isolation	4.63	2.59
Physical activity changes	6.08	2.28
Forming bonds with patients	6.34	2.29
Diet changes	6.54	1.90

Qualitative Results

The open-ended faculty responses identified some positive outcomes of the pandemic (Table 6). Major benefits included the utilization of remote learning and virtual platforms, the convenience of remote teaching, and the increased ability to participate in educational sessions. Faculty also appreciated increased flexibility related to remote meetings and decreased commute time. Faculty also appreciated the opportunity to teach and acquire new skills and knowledge, such as procedural skills, critical care medicine, and telehealth. Some of the challenges included a sense of emotional insecurity, a general decrease in wellness, concerns for safety, and a loss of personal connectivity with others. Faculty acknowledged that a work/life balance was increasingly difficult to obtain because of the lack of boundaries in the virtual environment.

**Table 6:** Faculty Qualitative Results

Benefits (79 total responses)	
Theme (% of responses)	Examples of Individual Responses
Remote (37%)	Flexibility with in-office vs at home administrative days
Convenience (22%)	Able to make multiple meetings more easily on a virtual platform
Participation (22%)	Able to lecture and log on from home - this has increased faculty participation since many of our faculty have a 40 min drive to the hospital.
Time (19%)	Less commuting for academic meetings allows this time to be utilized for other projects academic or personal; in general, meetings online allow for better work/life integration for those with families
Skills and knowledge (13%)	Increased opportunity for airway and other critical care procedures to do and teach; telemedicine
Challenges (114 total responses)	
Theme (% of responses)	Examples of Individual Responses
Emotional insecurity (28%)	Disruption, fear, anxiety around work, and risk to family
Wellness (27%)	Burnout; emotional draining
Safety (25%)	Stress of trying to care for/protect the residents; worry about bringing illness home to family; lack of protection - treated as expendable
Loss of personal connection (24%)	Feel distant from both work colleagues & family; lack of community; isolation and lack of community among colleagues (no in-person gathering)
Balance challenges (17%)	Zero free time - now that I can zoom into meetings, I am constantly working, even on days off; balancing management of residency through COVID-19 with the expectation that all other work will continue

## Discussion

As highlighted by the survey results, the pandemic’s influence on EM education was unprecedented, dynamic, and viewed in both a positive and negative light. Similar themes were reported at all levels of medical education and were demonstrated by both quantitative and qualitative measures. The transition to virtual platforms had positive and negative impacts on UME, GME, faculty, and wellness. The virtual platform allowed educators to innovate their curricula, leading to novel uses and opportunities for engagement with technology. Remote attendance at didactic conferences made participation easier for students, residents, and faculty; further, the remote attendance benefit extended beyond engagement in didactics as it allowed residents and faculty to more easily participate in departmental meetings and gatherings without the time commitment for travel.

However, many felt that the move to remote communication led to a decreased sense of connection with peers and colleagues resulting in a mixed picture of overall engagement and effectiveness. Other studies have shown that residents have decreased engagement with lectures in the online format, and often attendees are engaging in increased non-lecture-related activities during lecture time including email, entertainment, self-care, social, or other personal activities [13,14]. While evidence-based techniques for increasing engagement such as team-based or case-based learning may help, these have not yet been studied in the virtual didactic environment. In addition, although actual attendance or remote meetings and conferences is more convenient, many felt remote meetings led to a sense of personal disconnectedness, including the loss of in-person socialization and professional engagement.

Perhaps one area of connectedness that was increased in the virtual environment was the ability to meet one on one with students and residents for advising. Faculty noted that they felt it was easier to connect with students and provide them with more advice and mentorship via a virtual platform. Given the mixed picture of the use of virtual technologies for education and mentorship, it remains to be seen whether virtual platforms and educational uses of technology remain in place after the pandemic.

The impact of the pandemic on clinical education was particularly profound at the UME level, where students were pulled from rotations to mitigate exposure to infection, resulting in a loss of clinical and educational opportunities. However, the shift in environment gave our educators the opportunity to innovate their curriculum and incorporate many previously underutilized tools during the periods of most in-person restrictions. Many programs developed virtual rotations in EM, allowing for innovation resulting in an increase in medical knowledge as well as knowledge in areas not often explicitly taught on clerkships, such as social EM [15]. Despite these benefits, it is recognized that virtual rotations are still not an adequate substitute for clinical learning, with the long-term potential impacts yet to be recognized.

The disruption in the clinical environment also impacted residents, although to a lesser degree. While some residents had modified rotation schedules, they often were redeployed to other clinical areas rather than being pulled completely from clinical interactions with patients. The changes in resident clinical schedules were seen as a source of stress and anxiety, however, the impact on resident clinical education was mixed. Both benefits and challenges were seen within clinical experiences and resident skill and knowledge acquisition. While COVID-19 brought increased skills in airway management, critical care, and disaster medicine, it also led towards a decrease in the breadth of training experience through a loss of rotations and a loss of focus and exposure to other disease processes that are essential to EM trainees. Additionally, the clinical impact seemed to vary based on both location of programs and the phase of the pandemic.

Personal wellness suffered in all three groups, and feelings of safety and security were decreased overall during the pandemic. Disruptions to schedule and inability to connect personally with residents created difficulty for GME leaders to be able to recognize and help manage residents suffering from post-traumatic stress disorder (PTSD), depression, and burnout. While technology was deployed to try and promote camaraderie within programs and departments, it was felt to be a poor replacement for in-person communication and connection. Both residents and faculty appreciated having more time to spend at home due to the elimination of in-person events and meetings, and the time used for commuting was instead used to spend time with family or pursuing other interests, projects, and hobbies, which led to a better work/life integration. However, faculty noted that there was a limit to the convenience of blurring home/work boundaries that could potentially lead to issues of feeling unbalanced, disconnected, and burned out. At the UME level, the inability to forge personal connections led to a decreased understanding of the culture of residency programs, which was a source of stress for medical students during the application cycle. However, the use of the virtual platform created opportunities for students to more easily attend interviews and decrease travel costs during interview season, which was a clear benefit. As we transition away from many of the restrictions implemented, we need to consider a balance between the value of in-person interviewing with the benefits of virtual interviews.

Each of the surveyed groups developed different countermeasures to the challenges of the pandemic. For faculty, the top countermeasures were improving communication, offering remote education and meetings, and putting in place educational and clinical safety measures. These countermeasures were similar to those found in Rodriguez et al. reporting that enhanced availability of personal protective equipment (PPE), rapid and easy access to testing, and clear communication were keys to decreasing academic faculty, resident, and fellow stress and anxiety [16]. In our survey, both UME and GME respondents mentioned countermeasures focused on education interventions, including using technology to increase participation and remote learning for flexibility and safety with the knowledge that further study is needed to evaluate techniques to encourage deep learning and engagement in virtual didactic sessions. The GME survey respondents also noted an increased need to focus on wellness and clinical scheduling adjustments for safety and increased clinical coverage.

Countermeasures to mitigate impacts on wellness (across all survey groups) included offering mental health services, increased time at home, and a focus on teamwork and camaraderie. While attention to mental health and wellness was highlighted during the pandemic, other studies have shown that there were high levels of burnout and PTSD in EM residents pre-pandemic [17]. This suggests that educators should continue to focus on offering mental health services and screening for burnout, depression, and PTSD in EM trainees as the pandemic subsides.

Finally, when directly asked about the role of CORD during the pandemic, we found that respondents reported a variety of needs from CORD as an organizational body. The majority, regardless of their area of focus, looked for support and to provide educational resources. Examples of this included helping respondents to meet Accreditation Council for Graduate Medical Education (ACGME) guidelines and offering platforms to share ideas and innovative educational interventions. UME and GME educators looked to CORD to take on leadership and advocacy roles in the response to the impacts of the pandemic. Examples included an expectation for CORD to help provide recommendations, best practices, and collaboration opportunities among EM residency programs and other organizations.

The most important limitation of our findings is the small sample size. Although the survey links were sent out through various modalities and on several occasions, our response rates were low. This could be due to a combination of survey fatigue and overall stress and fatigue brought on by the pandemic. Both the small sample size and the specific responses gathered may also be impacted by the timing of the survey. The survey was deployed during the early pandemic, which may have affected responses in comparison to another phase, as situations and resources have been extremely dynamic throughout this time period. Although we attempted to describe the learning and work environment for all EM faculty, residents, and medical students, we recognize that this is an extremely heterogenous population and each region experienced very different impacts on learning environments, patient volumes, and percentage of COVID-19 patients. Finally, this survey was only filled out by EM educators (faculty/CORD members), who answered from their perspective, rather than the perspective of medical students and trainees themselves.

Recommendations for EM programs

1. Advocate at the institutional level for the ability to focus on the education of trainees without being compromised by service obligations to the hospital during times of increased clinical demand

2. Provide training for faculty and residents on the use of virtual platforms with a focus on engaged learning in adult learners

3. Focus on innovative ways to connect during periods of isolation

4. Improve access to mental health resources

5. Increase simulation experiences in response to decreased clinical exposure for students and residents

Recommendations for EM organizations including CORD

1. Support policies to ensure adequate protected time and residency funding to help train learners, regardless of clinical conditions

2. Develop standardized recommendations for clinician safety, including access to PPE, and establishment of appropriate workload during periods of increased clinical risk (for medical students, residents, faculty)

3. Advocate at the federal and state level for increased access to resources for hospitals facing disaster shortages (including materials and emergency staffing)

4. Develop core educational materials and form a centralized EM repository for sharing educational materials, especially those to be used in remote and/or asynchronous settings

5. Promote access to stress management, mental health, and resiliency resources and advocate for non-punitive utilization of mental health care

## Conclusions

The survey results highlighted the educational benefits and challenges faced by EM educators during the COVID-19 pandemic. Through the challenging times of the pandemic, many institutional and program-based innovations were developed and implemented to address the new educational environment. While individual endeavors were key to optimizing new educational models, there was a need for a coordinated effort within academic EM to examine these endeavors. This survey was intended to collate educational benefits and challenges, as well as creative and innovative approaches to the pandemic. On a national level, these approaches could provide invaluable educational tools for future training. Based on the common themes demonstrated in the survey data, the Task Force has developed a list of recommendations for best practices for EM programs and for EM organizations including CORD. We hope this information will prepare the EM academic community to respond to future educational disruptions similar to the COVID-19 pandemic.
